# The Effects of Endometriosis on Oocyte and Embryo Quality

**DOI:** 10.3390/jcm14072339

**Published:** 2025-03-28

**Authors:** Necati Findikli, Sandie Janssens, Giovanna Fasano, Isabelle Demeestere, Maxime Fastrez, Catherine Houba, Anne Delbaere

**Affiliations:** 1 Fertility Clinic, Department of Obstetrics and Gynecology, Université Libre de Bruxelles (ULB), Hôpital Universitaire de Bruxelles (H.U.B), CUB Hôpital Erasme, Route de Lennik 808, 1070 Brussels, Belgium; sandie.janssens@hubruxelles.be (S.J.); giovanna.fasano@hubruxelles.be (G.F.); isabelle.demeestere@hubruxelles.be (I.D.); maxime.fastrez@hubruxelles.be (M.F.); catherine.houba@hubruxelles.be (C.H.); anne.delbaere@hubruxelles.be (A.D.); 2 Research Laboratory on Human Reproduction, Université Libre de Bruxelles (ULB), Hôpital Universitaire de Bruxelles (H.U.B), CUB Hôpital Erasme, Route de Lennik 808, 1070 Brussels, Belgium

**Keywords:** endometriosis, oocyte quality, embryo development, infertility, assisted reproductive technologies

## Abstract

Endometriosis is a complex and multifaceted gynecological disorder characterized by the abnormal growth and presence of endometrial-like tissue outside the confines of the uterine cavity. It can lead to a wide range of distressing symptoms, including chronic pelvic pain, heavy and/or irregular menstrual bleeding, and significant challenges with fertility. While the association between endometriosis and infertility is well recognized, the precise mechanisms through which the disease affects oocyte and embryo quality remain controversial. Studies that utilized transcriptomic, metabolomic, and ultrastructural analyses indicated dysregulated energy metabolism, oxidative stress, mitochondrial dysfunction, and inflammatory alterations in the ovarian microenvironment. The impact of endometriosis on fertilization, embryo development, and implantation remains debated, with conflicting findings across different study designs. Some investigations reported impaired oocyte morphology, reduced fertilization rates, and poorer embryo quality, while others suggested that endometriosis does not significantly affect ART outcomes when confounding factors are controlled. Recent studies highlight the importance of distinguishing the disease severity, lesion location, and prior surgical interventions when assessing reproductive outcomes. The need for standardized methodologies in evaluating oocyte and embryo quality, alongside personalized treatment approaches, is emphasized. Further research is warranted to elucidate the precise molecular mechanisms underlying these effects and to develop targeted therapeutic strategies aimed at improving ART success in women with endometriosis. This narrative review provides a thorough examination of the previous research on the impact of endometriosis on oocyte and embryo quality, highlighting both the known mechanisms and the areas that require further investigation. This will help to guide future research and clinical management strategies to improve reproductive outcomes for women with endometriosis.

## 1. Introduction

Endometriosis is a common, benign, hormone-dependent, chronic gynecological disease associated with pelvic pain and infertility. The condition is characterized by the presence of endometrial-like tissue outside the uterine cavity, which can lead to various reproductive and non-reproductive complications. It affects approximately 10% of women of reproductive age and is associated with infertility in 25–50% of cases [1]. Despite its high prevalence, the exact mechanisms by which endometriosis impacts reproductive outcomes remain incompletely understood, and the ultimate clinical impact of the disease in both fertile and infertile populations is still largely unknown.

The ectopic endometrial tissue, responding to hormonal fluctuations, undergoes cyclical proliferation, shedding, and inflammation, mirroring the events within the uterus during menstruation [2]. This aberrant activity leads to a cascade of pathological consequences, including the formation of lesions, adhesions, and fibrosis in the affected areas, most commonly the pelvic peritoneum, ovaries, and rectovaginal septum. The resulting chronic inflammation and scarring contribute significantly to the hallmark symptoms of endometriosis, such as dysmenorrhea, dyspareunia, chronic pelvic pain, and irregular uterine bleeding [3].

Endometriosis can be classified into three main types: Superficial peritoneal endometriosis, the most common type, involves superficial lesions on the pelvic peritoneum, often appearing as small, punctate, or vesicular implants. Ovarian endometriosis, characterized by the formation of endometriomas or “chocolate cysts”, represents a more advanced stage of the disease, involving the invasion of the ovarian cortex and the accumulation of old blood within cystic structures. Deep infiltrating endometriosis, which is the most severe form, is defined by the presence of endometriotic lesions infiltrating more than 5 mm beneath the peritoneal surface, frequently affecting the rectovaginal septum, bowel, and bladder [4]. The disease also manifests in diverse forms, each characterized by distinct anatomical locations, histological features, and clinical presentations. So far, the publications in which the symptoms, analysis of biological samples, and/or genetics in endometriosis patients are reported mostly involve information regarding the stage of disease based on the widely adopted revised scoring system of the American Society for Reproductive Medicine (rASRM). This scoring system provides a score of I to IV (where I is “minimal” and IV is “severe”) based on the type, location, appearance, and depth of the invasion of the lesions and the extent of disease and adhesions [5].

The exact mechanisms driving the development and progression of endometriosis remain a subject of intense investigation, encompassing diverse theories ranging from the widely accepted retrograde menstruation hypothesis to the less understood concepts of coelomic metaplasia and lymphatic or hematogenous dissemination [6]. Endometriosis is also known to be inherited through generations, as the results of twin studies suggest that heritability may be as high as 50% [7]. The variable presentation of endometriosis, compounded by the lack of reliable non-invasive diagnostic tools, often leads to a delayed diagnosis and suboptimal management [8]. The gold standard for diagnosis remains surgical visualization and histological confirmation of endometriotic lesions, typically through laparoscopy. The location of lesions varies, as they can be found on the ovaries, fallopian tubes, and uterus. Ultrasonography can also help identify endometriotic cysts/lesions [9].

Management strategies for endometriosis usually aim to alleviate symptoms, improve quality of life, and preserve or restore fertility by encompassing a range of medical and surgical approaches. Medical therapies, including hormonal contraceptives, progestins, and gonadotropin-releasing hormone agonists, suppress ovarian hormone production, thereby inhibiting the growth and activity of endometriotic lesions. Surgical interventions, ranging from laparoscopic excision or the ablation of lesions to hysterectomy and oophorectomy in severe cases, aim to remove or destroy endometriotic tissue, alleviate pain, and restore pelvic anatomy [3]. While surgical treatment can be effective, complications associated with surgery may push the balance in favor of medical therapy. On the other hand, recent research indicates that alternative treatment modalities involving mesenchymal stem cells also show potential to be considered as viable therapeutic options in the near future [10].

There are several reviews in the literature indicating possible mechanisms by which endometriosis could exert its negative effects on the female reproductive system, as well as investigating the effect of the disease on oocytes, embryos, and treatment outcomes. Numerous researchers also previously investigated this relationship, but the results of their studies largely remained inconclusive, where some studies reported detrimental effects and others found no significant impact [11,12]. For women suffering from infertility, one of the primary concerns associated with endometriosis is its potential impact on the ovarian reserve, especially in the presence of endometrioma [13]. A reduced ovarian reserve can significantly affect the clinical outcome and success rates of Assisted Reproductive Technologies (ARTs). The impact of endometriosis on oocyte and embryo quality is still a matter of debate.

This review aims to synthesize the current understanding of the mechanisms by which endometriosis may influence oocyte and embryo quality, to delineate the reasons for conflicting laboratory and clinical outcomes for women with endometriosis, discuss potential research strategies to understand and document the true impact of the disease on oocytes and embryos.

## 2. Methodology

A search query in the PubMed database (National Library of Medicine, https://pubmed.ncbi.nlm.nih.gov/ accessed on 15 January 2025) performed with the time frame of January 2010 to December 2024 by using the search string “Endometriosis” AND (“In vitro fertilization” OR “Assisted Reproductive Technologies”) AND (“oocyte quality” OR “Oocyte morphology” OR “oocyte retrieval” OR “Oocyte yield”) resulted in 120 records published in the English language. To provide a robust and reliable synthesis of the available evidence, additional filtering was performed by the authors by focusing on more recent, relevant, and high-quality sources, including systematic reviews, large-scale observational studies, and meta-analyses. The results of the studies were analyzed and evaluated in more detail regarding the laboratory and clinical outcomes to establish a narrative review. In addition, several references not included in the search but considered important by the authors were also manually included in the evaluation.

## 3. Impact of Endometriosis on Follicular Development

Follicular development is the process through which a primary follicle develops within the somatic cells of the ovary into a specialized Graafian follicle with the potential to ovulate into the oviduct at mid-cycle. A typical growing ovarian follicle consists of an oocyte surrounded by several layers of cumulus cells (CCs) and mural granulosa cells (MGCs), theca cells (TCs), and a follicular antrum filled with follicular fluid (FF). CCs are connected to the oocyte by transzonal projections and are essential by acting as channels for nutrients and other important molecules to and from a developing oocyte [14]. MGCs and TCs are critical for maintaining the structural integrity of the follicle; delivering key metabolic components; and producing numerous important endocrine regulatory factors, such as androgens (testosterone and dihydrotestosterone), as well as growth regulatory factors (such as bone morphogenic proteins (BMPs) and transforming growth factor-β).

**Figure 1:**
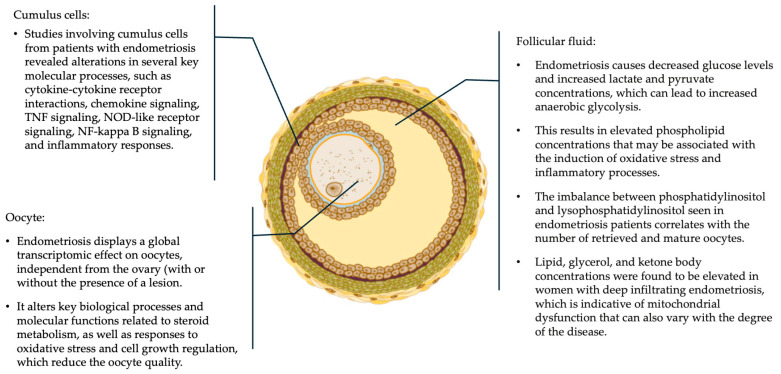
The possible effects of endometriosis on the molecular and cellular dynamics involved in oogenesis, as evidenced by transcriptomic and metabolomic studies.

The timely and efficient execution of molecular events and pathways through folliculogenesis is thus essential to produce a fertilization-competent oocyte, which can further create an implantation-competent embryo [15,16,17]. Findings from metabolomic studies regarding decreased glucose and increased lactate and pyruvate concentrations in FF indicate the presence of dysregulated energy metabolism and enhanced anaerobic glycolysis [18,19]. Increased oxidative stress and inflammation in the follicular environment can also lead to oxidative damage and impaired mitochondrial function in the oocytes [19]. Studies performed on CCs show that endometriosis can negatively affect follicular growth by creating dysregulations in the molecular pathways involving the CCs’ cell cycle and development, as well as alterations in the mitochondrial energy metabolism, steroid production, apoptosis, and inflammation [20,21]. The transcriptomic analyses of CCs from patients with endometriosis also revealed numerous differentially expressed genes compared with the controls, and the enrichment analysis evidenced altered molecular processes involving cytokine–cytokine receptor interactions, chemokine signaling, TNF signaling, NOD-like receptor signaling, NF-kappa B signaling, and inflammatory responses [22]. A comparison of the oocytes obtained from patients with endometriosis and healthy oocytes by whole-transcriptome profiling using scRNA-seq further resulted in a transcriptomic profile that was associated with lower oocyte quality in ovarian endometriosis [23]. The possible consequences of these adverse molecular changes through endometriosis on CCs, FF, and oocytes are summarized in Figure 1. Recent studies highlighted altered pro-inflammatory anti-inflammatory cytokines in endometriosis patients, which can be prognostic markers of oocyte and embryo quality in women with endometriosis [24]. An analysis of MII stage oocytes obtained from laparoscopically diagnosed endometriosis patients by transmission electron microscopy revealed increased mitochondrial structural abnormalities and reduced mitochondrial content [25]. Altered steroidogenesis also results in an imbalance of sex hormones and disruption of the optimal hormonal milieu required for oocyte maturation and development in endometriosis [26]. Aberrant angiogenesis and vascular function, as well as fibrosis, can, on the other hand, compromise the delivery of oxygen and nutrients to developing oocytes. The consequences of these ovarian microenvironmental changes can be extensive, potentially leading to a reduced follicular pool; impaired oocyte maturation; poor oocyte retrieval; and ultimately, compromised oocyte quality.

## 4. Impact of Endometriosis on Oocyte Quality

The oocyte quality can be defined in its simplest terms as the level of oocyte competency that can be assessed directly or indirectly by analyzing an oocyte’s morphology, as well as its nuclear and cytoplasmic maturation. Early studies that evaluated oocyte quality in endometriosis primarily focused on the number of oocytes collected and their maturity status during IVF (i.e., oocytes that cannot be used for insemination due to the lack of proper nuclear maturation) rather than on their detailed morphological characteristics [27,28]. Moreover, they were performed on fewer cases, and each evaluated only a limited number of oocyte morphological abnormalities. A possible effect of endometriosis on oocyte quality was documented in several studies involving oocyte donation treatments. When embryos obtained from donated oocytes from healthy women were transferred to women with endometriosis, the clinical outcomes (implantation and pregnancy rates) were found to be similar to patients without endometriosis. Likewise, in cycles where embryo transfers were performed by embryos generated from oocytes of women with stages III and IV endometriosis, the implantation rates were found to be significantly reduced, supporting the hypothesis that endometriosis can be associated with poor oocyte quality but not with endometrial receptivity [29,30]. Kasapoglu and her colleagues retrospectively investigated the impact of endometriosis on oocyte morphology in 72 patients and intracytoplasmic sperm injection outcomes and found that women with endometriosis in a clinical setting—particularly those with severe stages—tended to have poorer oocyte morphology compared with cases having male factor infertility. This included a higher incidence of cytoplasmic granularity, vacuoles, and zona pellucida abnormalities. While endometriosis did not appear to significantly affect fertilization rates, it might negatively impact the embryo quality and implantation rates in ICSI cycles. The authors suggest that oocyte morphological changes might also be attributed to oxidative stress and inflammation associated with endometriosis [31]. Shebl et al. conducted a matched case–control study to investigate the impact of endometriosis on oocyte competence and the subsequent IVF/ICSI treatment outcomes. Patients with endometriosis had a significantly lower rate of mature oocytes and an increased number of morphologically abnormal oocytes, particularly oocytes with darker cytoplasm and refractile bodies. They also found that the oocyte quality worsened with the increasing stage of endometriosis, indicating a stage-dependent and significant negative effect of endometriosis on ovarian physiology and disturbance in oogenesis, possibly via an increased mitochondrial dysfunction, as evidenced by the transcriptomic and metabolomic studies [18,22,23,32,33]. A more recent study by Robin and his colleagues examined the impact of endometriosis on oocyte morphology during IVF-ICSI procedures that involved a large cohort of over 6000 mature oocytes. Unlike the results of the previous studies, they found that endometriosis did not seem to have a significant impact on oocyte morphology in IVF-ICSI. Although the oocytes from the endometriosis group were more frequently misshapen and had intracytoplasmic vacuoles more often than those from the controls, the first polar body was found to be less often fragmented in this group [34]. Table 1 represents the studies and outcomes summary of the key published references to date.

## 5. Impact of Endometriosis on Fertilization and Embryo Quality

Successful fertilization, which can be defined as the proper union of sperm and oocyte nuclear material into a zygote, requires a rather timely and complex sequence of physiological events, such as hyperactivation, capacitation, acrosome reaction, and binding to the zona pellucida [40]. Studies showed that besides the effect of endometriosis on oocyte quality, it can also exert a negative effect on spermatozoa by altering the biochemical environment of the peritoneal cavity, which further decreases sperm function and sperm–oocyte interaction. Peritoneal fluid from endometriosis patients was found to contain elevated levels of reactive oxygen species (ROS), which reduced the sperm motility, viability, and zona pellucida binding capacity, and thus, created a potential negative effect on the fertilization rates [40,41]. Such potential should be considered as an additional negative contributing factor that can worsen the clinical outcome scenario in cases with advanced stages of the disease. From the laboratory outcome perspective, compared with conventional IVF (cIVF), the use of ICSI was initially proposed to overcome endometriosis-related fertilization problems and to increase the fertilization rates in non-male factor endometriosis cases [39]. Shebl and his colleagues reported a slightly reduced fertilization rate in the cIVF arm when compared with ICSI for the cases that were matched based on the method of fertilization [33]. However, Vigano and his colleagues, in their matched case-control study that involved 314 patients, documented that both cIVF and ICSI performed similarly in their clinical and laboratory settings [35].

The possible detrimental effects of endometriosis on oocyte quality have also been investigated through the quality and developmental potential of the resulting embryos. Embryo quality, which is the ability of an embryo to successfully implant and produce a live birth, is generally assessed via its static and morphokinetic developmental characteristics, which are mainly based on the number/size of the blastomeres, the degree of fragmentation at the cleavage stage and degree of expansion, and the trophectoderm/inner cell mass properties at the blastocyst stage. An in vitro study where human oocytes were exposed to endometriosis fluid from women with stage III/IV endometriosis reported a negative effect on the morphology of embryos with excess cellular fragmentation, proposing that increased cell fragmentation is implicated in impaired embryo development by inducing cell death in surrounding blastomeres or altering blastomere division [42].

In their recent systematic review and meta-analysis, Alseshre et al. found that the quantity and quality of the embryos produced were similar between patients with endometriosis and controls, although women with endometriomas who underwent IVF have been reported to have a significantly lower number of oocytes, as well as MII oocytes retrieved [43]. Likewise, in a large retrospective matched cohort study, Sánchez et al. investigated whether endometriosis negatively impacts embryo quality and development during IVF. They found no significant differences in the day 3 embryo quality (cell number, fragmentation, symmetry), fertilization rates, blastulation rates, or good/fair quality blastocyst formation between women with and without endometriosis. However, although the fertilization rate and number/quality of embryos were similar, the ongoing pregnancy rate was found to be reduced in the study group, possibly due to reduced endometrial receptivity or the limited value of the conventional embryo evaluation based on morphology [37]. In a very recent systematic review and meta-analysis by Gayete-Lafuente, patients with OMA exhibited similar fertilization, blastulation, and cancellation rates while displaying significantly lower numbers of total and mature (metaphase II) oocytes retrieved and lower numbers of top-quality embryos [44].

Besides studies that evaluated static embryo development characteristics, Cupino-Arcinue and colleagues evaluated five recently published studies to determine whether there exist significant disturbances in time-lapse embryo morphokinetics (TLM) that could predict the blastocyst quality, implantation, and success of pregnancy for embryos of women with endometriosis. The results showed overall inferior morphokinetic parameters of embryos when compared with the controls, independent of the severity of the endometriosis, yet possibly via the direct consequence of the compromised oocyte quality. Embryos with optimal early morphokinetic parameters, such as time to divide into 2 blastomeres (t2), time interval between cellular division from 3 to 4 blastomeres (S2), time of division into 5 blastomeres (t5) and late developmental events (such as compaction, morulation, time from insemination to start blastulation (tSB), and time from insemination to expanded blastocyst (tEB)) had better implantation rates than those who had suboptimal ranges. The authors pointed out that further studies are needed to determine whether using TLM for embryo selection in endometriosis improves pregnancy and live birth outcomes [45].

Junou et al. investigated the relationship between endometriosis and embryonic chromosomal abnormalities in in vitro fertilization patients by examining the rate of aneuploidy in age-matched cohorts after ART from women with and without endometriosis. Although previous in vitro research indicated an increase in the embryonic aneuploidy rates in oocytes exposed to an endometriotic environment, they reported that endometriosis did not significantly affect the aneuploidy rates in endometriosis patients in different female age groups [46]. The study further indicated that routine preimplantation genetic testing for aneuploidy may not be necessary for younger endometriosis patients undergoing IVF. Other factors, such as advanced maternal age or other underlying genetic conditions, might be more significant contributors to aneuploidy in endometriosis patients undergoing ART [47].

## 6. Impact of Endometriosis on Implantation and ART Outcomes

A successful pregnancy is preceded by an attachment of a developing blastocyst onto a receptive endometrium through a process called implantation. The implantation rate is therefore a common and indirect performance indicator of gamete and embryo quality/performance. In the matched case–control study by Shebl et al., it was further indicated that although there were notable differences in terms of maturation, oocyte quality, and fertilization rates, once the case reached the embryo transfer stage, no difference seemed to be observed concerning the rates of implantation, clinical pregnancy, miscarriage, live birth, and malformation in the patients with endometriosis [33]. In another matched case–control study, Wu et al. compared the laboratory and clinical outcomes of 1724 patients with or without ovarian endometrioma and reported that significantly lower oocyte maturation and fertilization rates, blastocyst rate, number of oocytes retrieved, and available embryos were found in women with endometrioma compared with the controls. Although the live birth rates were comparable between women with endometrioma and women in the control group, the cumulative live birth rate was found to be lower in the former group [36]. In their study, Sharma et al. [38] compared the IVF outcome of severe endometriosis cases with cases with tubal infertility. While the endometriosis group had higher levels of inflammatory markers and fewer mature eggs and quality embryos, these factors did not significantly impact the chances of a successful pregnancy in younger women (<35 years old). In contrast, endometriosis patients of age ≥ 35 years had a significantly lower likelihood of live birth and pregnancy rate when compared with the matched controls. This suggests that early diagnosis and intervention with ART can mitigate the potential negative effects of endometriosis on fertility outcomes in younger women [38].

Kamath and his colleagues recently investigated whether endometriosis affects live birth rates following donor oocyte recipient versus autologous IVF cycles by utilizing a large registry of the Human Fertilization and Embryology Authority (HFEA) data from the UK [48]. The results indicate that there was no significant difference in the live birth rates between women with endometriosis who underwent donor oocyte recipient cycles and those who underwent autologous IVF cycles. While the LBR in women with endometriosis undergoing donor oocyte recipient cycles was slightly lower compared with women without endometriosis undergoing the same procedure (28.0% vs. 30.7%), this difference was not statistically significant, suggesting that impaired endometrial receptivity, rather than oocyte quality, could be a primary factor influencing poorer IVF outcomes in women with endometriosis. In a recent systematic review and meta-analysis where only studies in which women with endometriosis had not undergone any previous surgical or medical treatments for this condition were selected, similar fertilization but a reduced implantation rate was observed in cases with endometriosis compared with patients having other infertility indications [49]. Similarly, results of another recent systematic review and meta-analysis using two large registries (7212 oocyte donation cycles from published studies and 162,082 cycles from SART and HFEA registries) suggest that uterine receptivity and implantation in patients with a history of endometriosis undergoing vitro fertilization may be marginally affected since both analyses revealed a modest reduction in live birth rates among recipients with endometriosis [50]. Such a reduction could also be related to impaired endometrial receptivity, altered adhesion molecule expression, and autophagy in adenomyosis since studies indicate that adenomyosis frequently coexists in patients with endometriosis [51].

## 7. Oocyte Quality in Endometriosis: Where Do We Go from Here?

The findings presented in the current literature regarding the possible effects of endometriosis on oocyte and embryo quality are still controversial, highlighting the multifactorial nature of reproductive challenges associated with endometriosis. While possible mechanisms on how endometriosis can affect the oocyte quality and embryo development are well-documented by several groups worldwide, more research, both in vitro and clinical, is needed to further elucidate the complex network of the molecular pathways involved and to elaborate on which conditions this impact can be predicted or managed by available treatment modalities. The contemporary management of endometriosis is nowadays shaped by the severity of symptoms, location and extent of the disease, age of the patient, and the status/desire for fertility. Current treatment approaches mainly encompass surgical interventions; various drug therapies, including hormone therapy; and the use of anti-inflammatory agents.

In the presence of endometriosis-related infertility, special attention must be paid to tailor the treatment by both alleviating patient discomfort while preserving fertility and improving the outcome with ART. For patients undergoing fertility preservation by oocyte vitrification, current data indicate similar survival rates, albeit a significantly negative effect of previous surgery in terms of the number of oocytes retrieved during the OPU procedure [52]. Although reduced inflammation, improved oocyte quality, and restored endometrial receptivity after its use were recently postulated, data regarding the possible positive effects of the administration of a GnRH agonist before IVF are scarce and such outcomes should be documented by well-designed clinical trials regarding its role [53].

Understanding how endometriosis impacts oocyte quality can therefore help enhance treatment outcomes by reducing oocyte damage and ultimately improving the clinical pregnancy and live birth rates. Although several emerging therapeutic strategies, such as vitamins and/or antioxidant supplementation, as well as immune-modulating therapies, can show promise in mitigating the negative effects that can be caused by endometriosis, their possible impacts are still considered controversial and under investigation [54]. Recent studies also indicate that oocyte donation could be a valid option for certain patients with endometriosis experiencing multiple IVF failures, where the psychosocial status of the patients and the legal framework of the country are also suitable [30].

A recent study conducted by Somigliana and his colleagues took a fresh and possibly more accurate approach to examining the connection between endometriosis and clinical outcomes in ART, including oocyte and embryo quality. The authors meticulously analyzed each phase of the IVF process, emphasizing intra-patient comparisons, which are particularly revealing for unilateral endometriomas, as well as matched studies. Their findings suggest that the presence of endometriosis may not have an impact on the ovarian response. The quality of oocytes appeared to be maintained, and follicular steroidogenesis did not show any adverse effects in women with endometriosis [55]. On the other hand, it has been reported that the ovarian response was notably diminished when endometriomas exceed 4 cm in diameter, indicating that disease-specific parameters make the current comparisons very complex [56]. Moreover, since the fertilization rates were comparable with those observed in individuals without endometriosis, indicating that invasive fertilization techniques, such as ICSI (intracytoplasmic sperm injection), may not be necessary. Additionally, embryological development was found to be similar, with no rise in aneuploidy rates [55].

Recent publications also indicate that in most of the previously published studies in which significant negative effects on the laboratory and clinical outcomes have been reported, the presence of adenomyosis, as well as the presence of previous endometriotic surgeries, may act as major confounding factors that create significant bias due to operation-related morbidities [51,57]. Moreover, the considerable heterogeneity in terms of the study design, inclusion/exclusion criteria, confounding endocrinological disturbances that remain undetected (i.e., thyroid autoimmunity) due to incomplete infertility workup, patient/disease-specific characteristics, and oocyte/embryo assessment approaches exist in the available literature, making the evaluation and interpretation of the results extremely complex [58]. Although still controversial, current results reinforce the idea that endometriosis per se may not significantly increase the risk of poor oocyte quality, impair embryo development, and increase implantation failure in all cases with endometriosis. It, however, indicates that the management and treatment of women with endometriosis require more individualized, holistic, and multidisciplinary approaches according to patients’ needs [59].

## 8. Conclusions

Studies that investigated the pathogenesis of endometriosis indicate that it can affect gamete and embryo quality through several disturbed and/or dysregulated molecular pathways involving oxidative stress, inflammation, apoptosis, and altered gene expression. Addressing these challenges requires a multidisciplinary approach, integrating advanced diagnostic tools and personalized therapeutic interventions to increase the outcome by increasing the oocyte and embryo quality. Future research should also focus on understanding the molecular mechanisms underlying these effects, developing more objective and noninvasive oocyte and embryo assessment tools, and developing targeted therapies to improve reproductive outcomes. While the previously published literature indicates that endometriosis can have an impact on the oocyte and embryo quality, new studies suggest that the relationship between endometriosis and IVF outcomes may be more complex than once considered and can easily be misinterpreted due to a lack of standardization in the study settings, as well as a limited number of patient/cases. Nevertheless, the presence of endometriosis, even in minimal or mild forms, can still be associated with reduced MII oocyte retrieval due to a reduced ovarian reserve, decreased oocyte quality, increased rates of apoptosis, and impaired embryo development in some cases. These findings highlight the importance of early diagnosis and appropriate management of endometriosis to optimize outcomes. Understanding the underlying mechanisms better, discovering novel and more effective (early, type/stage-specific, non-invasive) cellular and molecular markers, and developing targeted therapies to address these issues remain crucial areas of research to improve the outcomes of ART for women with endometriosis.

## Figures and Tables

**Table 1:** Summary of cited studies with respect to their impacts on oocyte, fertilization, and embryo quality.

Cited Reference	Study Design	Num. of Patients		Key Findings
		Endometriosis	Control	
Vigano et al., 2023 [35]	Matched case–control study	157	157	The fertilization rate and the proportion of women with total fertilization failure did not differ between the groups.
Wu et al., 2021 [36]	Matched case–control study	862	862	Significantly lower oocyte maturation, number of oocytes retrieved, fertilization rate, blastocyst rate, and number of available embryos were found in women with endometrioma compared with the control.
Robin et al., 2021 [34]	Retrospective comparative study	175	401	No difference in oocyte quality (based on AOQI and MOMS scores) was found between the groups. Increased rate of oocyte morphological abnormalities (abnormal shape, perivitelline debris, cytoplasmic vacuoles) was observed in the endometriosis group.
Sanchez et al., 2020 [37]	Matched case–control study	429	851	The fertilization rate was found to be similar between the groups. Quality of embryos at the cleavage and blastocyst stages were similar between the groups.
Sharma et al., 2020 [38]	Matched case–control study	294	358	Number of mature oocytes and good-quality embryos were observed to be significantly lower in women with endometriosis when compared with the matched controls.
Shebl et al., 2017 [33]	Matched case–control study	119	114	Oocyte quality and maturation rate were reduced in the endometriosis arm. Increased rate of oocyte morphologically abnormalities (dark cytoplasm, refractile bodies) were observed in the endometriosis group. A significantly higher fertilization rate was found in the ICSI group compared with the IVF group in endometriosis patients.
Kasapoglu et al., 2017 [31]	Retrospective cohort study	72	60	Oocyte quality was found to be reduced. Increased rate of oocyte morphological abnormalities (dark cytoplasm; dark, large, or thin zona pellucida; flat or fragmented polar body) were observed in the endometriosis group.
Goud et al., 2014 [28]	Prospective cohort study	10	18	Oocyte quality was found to be reduced. Increased rate of oocyte morphological abnormalities (spindle disruption, higher ZP dissolution timing) was observed in the endometriosis group.
Komsky-Elbaz et al., 2013 [39]	Prospective randomized trial	35	-	A significantly higher fertilization rate was found in the ICSI group compared with the cIVF group.

## Data Availability

Not applicable.

## References

[1] Zondervan K.T., Becker C.M., Missmer S.A. (2020). Endometriosis. N. Engl. J. Med..

[2] Camboni A., Marbaix E. (2021). Ectopic Endometrium: The Pathologist’s Perspective. Int. J. Mol. Sci..

[3] Saunders P.T.K., Horne A.W. (2021). Endometriosis: Etiology, Pathobiology, and Therapeutic Prospects. Cell.

[4] Imperiale L., Nisolle M., Noël J.-C., Fastrez M. (2023). Three Types of Endometriosis: Pathogenesis, Diagnosis and Treatment. State of the Art. J. Clin. Med..

[5] American Society For Reproductive Medicine (1997). Revised American Society for Reproductive Medicine Classification of Endometriosis: 1996. Fertil. Steril..

[6] Griffiths M.J., Horne A.W., Gibson D.A., Roberts N., Saunders P.T.K. (2024). Endometriosis: Recent Advances That Could Accelerate Diagnosis and Improve Care. Trends Mol. Med..

[7] Lalami I., Abo C., Borghese B., Chapron C., Vaiman D. (2021). Genomics of Endometriosis: From Genome Wide Association Studies to Exome Sequencing. Int. J. Mol. Sci..

[8] Saunders P.T.K., Whitaker L.H.R., Horne A.W. (2024). Endometriosis: Improvements and Challenges in Diagnosis and Symptom Management. Cell Rep. Med..

[9] Tan S., Leonardi M., Lo G., Lee E. (2024). Role of Ultrasonography in the Diagnosis of Endometriosis in Infertile Women: Ovarian Endometrioma, Deep Endometriosis, and Superficial Endometriosis. Best Pract. Res. Clin. Obstet. Gynaecol..

[10] Chatzianagnosti S., Dermitzakis I., Theotokis P., Kousta E., Mastorakos G., Manthou M.E. (2024). Application of Mesenchymal Stem Cells in Female Infertility Treatment: Protocols and Preliminary Results. Life.

[11] Dongye H., Tian Y., Qi D., Du Y., Yan L. (2023). The Impact of Endometrioma on Embryo Quality in In Vitro Fertilization: A Retrospective Cohort Study. J. Clin. Med..

[12] Casalechi M., Di Stefano G., Fornelli G., Somigliana E., Viganò P. (2024). Impact of Endometriosis on the Ovarian Follicles. Best Pract. Res. Clin. Obstet. Gynaecol..

[13] Henry L., Vervier J., Boucher A., Brichant G., Gaspard O., Labied S., Munaut C., Ravet S., Nisolle M. (2022). Oocyte Cryopreservation in Patients with Endometriosis: Current Knowledge and Number Needed to Treat. J. Clin. Med..

[14] Clarke H.J. (2022). Transzonal Projections: Essential Structures Mediating Intercellular Communication in the Mammalian Ovarian Follicle. Mol. Reprod. Dev..

[15] Eppig J.J. (2018). Reproduction: Oocytes Call, Granulosa Cells Connect. Curr. Biol..

[16] Young J.M., McNeilly A.S. (2010). Theca: The Forgotten Cell of the Ovarian Follicle. Reproduction.

[17] Oktem O., Urman B. (2010). Understanding Follicle Growth In Vivo. Hum. Reprod..

[18] Kobayashi H., Imanaka S. (2024). Recent Progress in Metabolomics for Analyzing Common Infertility Conditions That Affect Ovarian Function. Reprod. Med. Biol..

[19] Pan Y., Pan C., Zhang C. (2024). Unraveling the Complexity of Follicular Fluid: Insights into Its Composition, Function, and Clinical Implications. J. Ovarian Res..

[20] Fan W., Yuan Z., Li M., Zhang Y., Nan F. (2023). Decreased Oocyte Quality in Patients with Endometriosis Is Closely Related to Abnormal Granulosa Cells. Front. Endocrinol..

[21] Simopoulou M., Rapani A., Grigoriadis S., Pantou A., Tsioulou P., Maziotis E., Tzanakaki D., Triantafyllidou O., Kalampokas T., Siristatidis C. (2021). Getting to Know Endometriosis-Related Infertility Better: A Review on How Endometriosis Affects Oocyte Quality and Embryo Development. Biomedicines.

[22] Da Luz C.M., Da Broi M.G., Koopman L.D.O., Plaça J.R., Da Silva-Jr W.A., Ferriani R.A., Meola J., Navarro P.A. (2022). Transcriptomic Analysis of Cumulus Cells Shows Altered Pathways in Patients with Minimal and Mild Endometriosis. Sci. Rep..

[23] Ferrero H., Corachán A., Aguilar A., Quiñonero A., Carbajo-García M.C., Alamá P., Tejera A., Taboas E., Muñoz E., Pellicer A. (2019). Single-Cell RNA Sequencing of Oocytes from Ovarian Endometriosis Patients Reveals a Differential Transcriptomic Profile Associated with Lower Quality. Hum. Reprod..

[24] Singh A.K., Dutta M., Chattopadhyay R., Chakravarty B., Chaudhury K. (2016). Intrafollicular Interleukin-8, Interleukin-12, and Adrenomedullin Are the Promising Prognostic Markers of Oocyte and Embryo Quality in Women with Endometriosis. J. Assist. Reprod. Genet..

[25] Xu B., Guo N., Zhang X., Shi W., Tong X., Iqbal F., Liu Y. (2015). Oocyte Quality Is Decreased in Women with Minimal or Mild Endometriosis. Sci. Rep..

[26] Sanchez A.M., Somigliana E., Vercellini P., Pagliardini L., Candiani M., Vigano P. (2016). Endometriosis as a Detrimental Condition for Granulosa Cell Steroidogenesis and Development: From Molecular Alterations to Clinical Impact. J. Steroid Biochem. Mol. Biol..

[27] Sanchez A.M., Vanni V.S., Bartiromo L., Papaleo E., Zilberberg E., Candiani M., Orvieto R., Viganò P. (2017). Is the Oocyte Quality Affected by Endometriosis? A Review of the Literature. J. Ovarian Res..

[28] Goud P.T., Goud A.P., Joshi N., Puscheck E., Diamond M.P., Abu-Soud H.M. (2014). Dynamics of Nitric Oxide, Altered Follicular Microenvironment, and Oocyte Quality in Women with Endometriosis. Fertil. Steril..

[29] Latif S., Saridogan E. (2023). Endometriosis, Oocyte, and Embryo Quality. J. Clin. Med..

[30] Maignien C., Hachem R.E., Bourdon M., Marcellin L., Chalas C., Patrat C., Gonzàlez-Foruria I., Chapron C., Santulli P. (2023). Oocyte Donation Outcomes in Endometriosis Patients with Multiple IVF Failures. Reprod. Biomed. Online.

[31] Kasapoglu I., Kuspinar G., Saribal S., Turk P., Avcı B., Uncu G. (2018). Detrimental Effects of Endometriosis on Oocyte Morphology in Intracytoplasmic Sperm Injection Cycles: A Retrospective Cohort Study. Gynecol. Endocrinol. Off. J. Int. Soc. Gynecol. Endocrinol..

[32] Tatíčková M., Trebichalská Z., Kyjovská D., Otevřel P., Kloudová S., Holubcová Z. (2023). The Ultrastructural Nature of Human Oocytes’ Cytoplasmic Abnormalities and the Role of Cytoskeleton Dysfunction. F&S Sci..

[33] Shebl O., Sifferlinger I., Habelsberger A., Oppelt P., Mayer R.B., Petek E., Ebner T. (2017). Oocyte Competence in In Vitro Fertilization and Intracytoplasmic Sperm Injection Patients Suffering from Endometriosis and Its Possible Association with Subsequent Treatment Outcome: A Matched Case-Control Study. Acta Obstet. Gynecol. Scand..

[34] Robin C., Uk A., Decanter C., Behal H., Collinet P., Rubod C., Barbotin A.-L., Robin G. (2021). Impact of Endometriosis on Oocyte Morphology in IVF-ICSI: Retrospective Study of a Cohort of More than 6000 Mature Oocytes. Reprod. Biol. Endocrinol..

[35] Viganò P., Reschini M., Ciaffaglione M., Cucè V., Casalechi M., Benaglia L., Vercellini P., Somigliana E. (2023). Conventional IVF Performs Similarly in Women with and Without Endometriosis. J. Assist. Reprod. Genet..

[36] Wu Y., Yang R., Lan J., Lin H., Jiao X., Zhang Q. (2021). Ovarian Endometrioma Negatively Impacts Oocyte Quality and Quantity But Not Pregnancy Outcomes in Women Undergoing IVF/ICSI Treatment: A Retrospective Cohort Study. Front. Endocrinol..

[37] Sanchez A.M., Pagliardini L., Cermisoni G.C., Privitera L., Makieva S., Alteri A., Corti L., Rabellotti E., Candiani M., Viganò P. (2020). Does Endometriosis Influence the Embryo Quality and/or Development? Insights from a Large Retrospective Matched Cohort Study. Diagnostics.

[38] Sharma S., RoyChoudhury S., Bathwal S., Bhattacharya R., Kalapahar S., Chattopadhyay R., Saha I., Chakravarty B. (2020). Pregnancy and Live Birth Rates Are Comparable in Young Infertile Women Presenting with Severe Endometriosis and Tubal Infertility. Reprod. Sci..

[39] Komsky-Elbaz A., Raziel A., Friedler S., Strassburger D., Kasterstein E., Komarovsky D., Ron-El R., Ben-Ami I. (2013). Conventional IVF Versus ICSI in Sibling Oocytes from Couples with Endometriosis and Normozoospermic Semen. J. Assist. Reprod. Genet..

[40] Gadella B.M., Luna C. (2014). Cell Biology and Functional Dynamics of the Mammalian Sperm Surface. Theriogenology.

[41] Sáez-Espinosa P., Velasco I., Lorca P., Acién M.I., Romero A., Gómez-Torres M.J. (2020). Peritoneal Fluid from Women with Endometriosis Impairs Human Spermatozoa Functionality. Reprod. Biol..

[42] Paffoni A., Bolis V., Ferrari S., Benaglia L., Vercellini P., Somigliana E. (2019). The Gametotoxic Effects of the Endometrioma Content: Insights From a Parthenogenetic Human Model. Reprod. Sci..

[43] Alshehre S.M., Narice B.F., Fenwick M.A., Metwally M. (2021). The Impact of Endometrioma on In Vitro Fertilisation/Intra-Cytoplasmic Injection IVF/ICSI Reproductive Outcomes: A Systematic Review and Meta-Analysis. Arch. Gynecol. Obstet..

[44] Gayete-Lafuente S., Vilà Famada A., Albayrak N., Espinós Gómez J.J., Checa Vizcaíno M.Á., Moreno-Sepulveda J. (2024). Indirect Markers of Oocyte Quality in Patients with Ovarian Endometriosis Undergoing IVF/ICSI: A Systematic Review and Meta-analysis. Reprod. Biomed. Online.

[45] Cupino-Arcinue D., Seeber B., Montag M., Toth B. (2024). Does Endometriosis Inflict Harm on Embryos? A Systematic Review of Embryo Morphokinetics Analysed by Time Lapse Monitoring in Women with Endometriosis. Arch. Gynecol. Obstet..

[46] Juneau C., Kraus E., Werner M., Franasiak J., Morin S., Patounakis G., Molinaro T., de Ziegler D., Scott R.T. (2017). Patients with Endometriosis Have Aneuploidy Rates Equivalent to Their Age-Matched Peers in the In Vitro Fertilization Population. Fertil. Steril..

[47] Qu H., Lv H., Kang Y., Yan L., Du Y. (2024). Reproductive Outcomes of Single Frozen-Thawed Embryo Transfer in Patients with Endometriosis after Preimplantation Genetic Testing. J. Assist. Reprod. Genet..

[48] Kamath M.S., Subramanian V., Antonisamy B., Sunkara S.K. (2022). Endometriosis and Oocyte Quality: An Analysis of 13 614 Donor Oocyte Recipient and Autologous IVF Cycles. Hum. Reprod. Open.

[49] Mappa I., Page Z.P., Di Mascio D., Patelli C., D’Antonio F., Giancotti A., Gebbia F., Mariani G., Cozzolino M., Muzii L. (2024). The Effect of Endometriosis on In Vitro Fertilization Outcomes: A Systematic Review and Meta-Analysis. Healthcare.

[50] Paffoni A., Casalechi M., De Ziegler D., Cicinelli E., Somigliana E., Viganò P., Vitagliano A. (2024). Live Birth After Oocyte Donation In Vitro Fertilization Cycles in Women with Endometriosis: A Systematic Review and Meta-Analysis. JAMA Netw. Open.

[51] Wu H.-M., Tsai T.-C., Liu S.-M., Pai A.H.-Y., Chen L.-H. (2024). The Current Understanding of Molecular Mechanisms in Adenomyosis-Associated Infertility and the Treatment Strategy for Assisted Reproductive Technology. Int. J. Mol. Sci..

[52] Cobo A., Giles J., Paolelli S., Pellicer A., Remohí J., García-Velasco J.A. (2020). Oocyte Vitrification for Fertility Preservation in Women with Endometriosis: An Observational Study. Fertil. Steril..

[53] Panagodimou E.K., Kalogeropoulos S., Adonakis G., Kaponis A. (2024). Does Gonadotropin-Releasing Hormone Agonist Administration Before Assisted Reproduction Techniques Improve Pregnancy Rates in Women With Endometriosis?. Obstet. Gynecol. Surv..

[54] Corachán A., Pellicer N., Pellicer A., Ferrero H. (2021). Novel Therapeutic Targets to Improve IVF Outcomes in Endometriosis Patients: A Review and Future Prospects. Hum. Reprod. Update.

[55] Somigliana E., Li Piani L., Paffoni A., Salmeri N., Orsi M., Benaglia L., Vercellini P., Vigano’ P. (2023). Endometriosis and IVF Treatment Outcomes: Unpacking the Process. Reprod. Biol. Endocrinol..

[56] Somigliana E., Palomino M.C., Castiglioni M., Mensi L., Benaglia L., Vercellini P., Garcia-Velasco J. (2020). The Impact of Endometrioma Size on Ovarian Responsiveness. Reprod. Biomed. Online.

[57] Bourdon M., Peigné M., Maignien C., de Villardi de Montlaur D., Solignac C., Darné B., Languille S., Bendifallah S., Santulli P. (2024). Impact of Endometriosis Surgery on In Vitro Fertilization/Intracytoplasmic Sperm Injection Outcomes: A Systematic Review and Meta-Analysis. Reprod. Sci..

[58] Salmeri N., Gennarelli G., Vanni V.S., Ferrari S., Ruffa A., Rovere-Querini P., Pagliardini L., Candiani M., Papaleo E. (2023). Concomitant Autoimmunity in Endometriosis Impairs Endometrium-Embryo Crosstalk at the Implantation Site: A Multicenter Case-Control Study. J. Clin. Med..

[59] Trinchant R., García-Velasco J.A. (2024). Oocyte Quality in Women with Endometriosis. Gynecol. Obstet. Investig..

